# In Vitro Biological Effects of E-Cigarette on the Cardiovascular System—Pro-Inflammatory Response Enhanced by the Presence of the Cinnamon Flavor

**DOI:** 10.3390/toxics10120784

**Published:** 2022-12-14

**Authors:** Marine Michon, Clément Mercier, Claudie Petit, Lara Leclerc, Laurent Bertoletti, Jérémie Pourchez, Valérie Forest

**Affiliations:** 1 Mines Saint-Etienne, Univ Jean Monnet, INSERM, U1059 Sainbiose, Centre CIS, 42023 Saint-Etienne, France; 2 Service de Médecine Vasculaire et Thérapeutique, CHU de Saint-Etienne, 42055 Saint-Etienne, France; 3 INSERM, UMR1059, Equipe Dysfonction Vasculaire et Hémostase, Université Jean-Monnet, 42055 Saint-Etienne, France; 4 INSERM, CIC-1408, CHU Saint-Etienne, 42055 Saint-Etienne, France

**Keywords:** e-cigarette, toxicity, cardiovascular damage, pro-inflammatory response, e-cigarette aerosol condensate, nicotine, flavor, vaping

## Abstract

The potential cardiovascular effects of e-cigarettes remain largely unidentified and poorly understood. E-liquids contain numerous chemical compounds and can induce exposure to potentially toxic ingredients (e.g., nicotine, flavorings, etc.). Moreover, the heating process can also lead to the formation of new thermal decomposition compounds that may be also hazardous. Clinical as well as in vitro and in vivo studies on e-cigarette toxicity have reported potential cardiovascular damages; however, results remain conflicting. The aim of this study was to assess, in vitro, the toxicity of e-liquids and e-cigarette aerosols on human aortic smooth muscle cells. To that purpose, cells were exposed either to e-liquids or to aerosol condensates obtained using an e-cigarette device at different power levels (8 W or 25 W) to assess the impact of the presence of: (i) nicotine, (ii) cinnamon flavor, and (iii) thermal degradation products. We observed that while no cytotoxicity and no ROS production was induced, a pro-inflammatory response was reported. In particular, the production of IL-8 was significantly enhanced at a high power level of the e-cigarette device and in the presence of the cinnamon flavor (confirming the suspected toxic effect of this additive). Further investigations are required, but this study contributes to shedding light on the biological effects of vaping on the cardiovascular system.

## 1. Introduction

Vaping devices (electronic cigarettes or e-cigarettes) are considered by some public health agencies and scientists as a helpful tool for smoking cessation and a harm reduction strategy in the public health practice of tobacco control [1,2]. While many studies tend to show that vaping is intrinsically less hazardous and, thus, most certainly safer than conventional smoking tobacco products, that does not mean e-cigarettes are completely innocuous. In particular, further investigations are warranted to better characterize the long-term cardiovascular effects of the aerosol generated by e-cigarettes from the nicotine-containing fluids [3]. Indeed, instead of burning tobacco, the e-cigarette allows the heating of a so-called e-liquid, generating an aerosol that is inhaled by the user. The formulation of e-liquids involves up to several dozen chemical compounds and includes, for instance, propylene glycol, glycerol, water, ethanol, flavorings, and, in most cases, nicotine. Some of these ingredients can exhibit an intrinsic toxicity, but the heating process of the e-liquid can also lead to the formation of new thermal decomposition compounds in emissions that may also be hazardous [3,4,5]. Approximately 250 chemical substances have already been detected in vaping emissions, including substances initially present as ingredients in the e-liquid formulation (e.g., nicotine, flavorings, propylene glycol), but also a large number of thermal degradation products present in the gas phase generated by e-cigarettes (e.g., alkaloids, volatile organic compounds (VOCs), pyridine, carbonyl compounds such as acrolein or formaldehyde). Concentration of thermal degradation products are voltage-dependent [6]. Metal particles (such as iron, aluminum, chromium, nickel) have also been previously found in relatively high concentrations in e-cigarette emissions [4,7,8]. Indeed, heating coils used in e-cigarette atomizers can generate evaporated metals (this generation increases when the electrical power applied increases), which then coagulate into metallic (nano)particle clusters.

While many studies focus on the toxicity of e-cigarette on the respiratory tract, as lungs are the first target of inhaled aerosols produced by e-cigarettes, cardiovascular damages have also been reported. They include vascular endothelial damage, endothelial function impairment, arterial stiffness, increased oxidative stress, acute effects on heart rate and blood pressure, and long-term risk for coronary events [9,10,11,12,13,14,15,16]. In vitro and in vivo preclinical studies have shown that exposure to e-cigarette emissions or extracts resulted in genotoxic effects such as DNA damage or inhibition of DNA repair [16]. Exposure to e-cigarette aerosol increases vascular, cerebral, and pulmonary oxidative stress [17] and is also associated with inflammation [6]. E-cigarettes were also reported to cause short-term effects on platelet function, increasing platelet activation and aggregation [18]. Platelet and leukocyte activation as well as endothelial dysfunction are associated with atherogenesis and cardiovascular morbidity [19,20]. However, many results reported in the literature remain conflicting, suggesting that the potential cardiovascular consequences of e-cigarettes require further investigation.

The aim of this study was to assess, in vitro, the toxicity of different formulations of e-liquids and condensates of e-cigarette emissions on human aortic smooth muscle cells. We chose this cell model because, although not directly in contact with blood, these cells regulate actomyosin involved in contraction and are partly responsible for arterial stiffening, which is an observed clinical effect of e-cigarettes [21,22]. Concretely, cells have been exposed either to e-liquid (with or without nicotine) or to the aerosol condensate obtained using e-cigarettes with different power levels (low: 8 W or high: 25 W) to assess the impact of thermal degradation products. The impact of the presence of the cinnamon flavor, a common additive to e-liquids known to exhibit a toxic effect [23], has also been investigated.

## 2. Materials and Methods

### 2.1. Cell Culture

Human aortic smooth muscle cells (AoSMC) were purchased from Lonza (Basel, Switzerland) and maintained in Smooth Muscle Cell Growth Medium-2 (SmGM, Lonza, Basel, Switzerland). Cells were differentiated for one to two weeks in Smooth Muscle Cell Basal Medium (SmBM, Lonza, Basel, Switzerland) added with 5% penicillin/streptomycin (VWR, Monroeville, PA, USA). The culture was carried out at 37 °C in 5% CO_2_ humidified atmosphere. Culture medium was changed twice or three times per week. The flasks were subcultured with trypsin (0.025%) /EDTA (Lonza, Basel, Switzerland) when cells reached around 80% confluence. Then, 96-well plates were coated with 3 µg/cm² fibronectin (Sigma-Merck, Saint-Quentin-Fallavier, France), reconstituted with 1 mL of phosphate buffered saline (PBS, Fisher Scientific, Illkirch, France). Plates were then air-dried for 45 min and stored for 2–4 weeks. A total of 15,000 cells in 50 µL of medium (SmBM, Lonza, Basel, Switzerland) were plated in each well and incubated at 37 °C under a 5% CO_2_ atmosphere. Cells were allowed to adhere for 24 h (cells reached about 80% confluence). Then, 150 µL of culture medium, in which was diluted either the e-liquid or the aerosol condensate (within the 2–128 µg/mL concentration range), were added to reach a final volume of 200 µL in each well. The cells were then exposed for 24 h, a common duration for this type of exposure.

### 2.2. E-liquids and Aerosol Condensates Preparation

**Figure 1:**
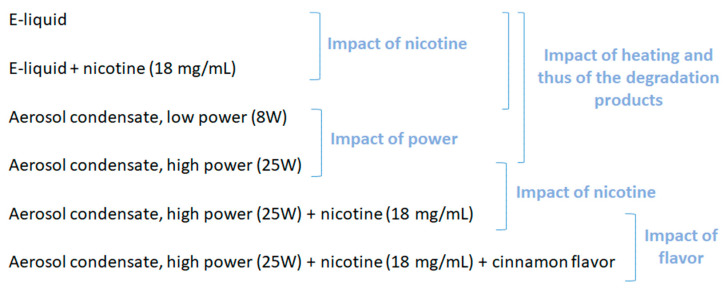
Definition of the experimental conditions used in this study and the comparisons that allowed determination of the impact of different parameters.

E-liquids and nicotine were purchased from A&L (Arômes et Liquides, Andrézieux-Bouthéon, France: vegetable base liquid 50/50 PG/VG (Propylene Glycol/ Vegetable Glycerin) and A&L booster 20 mg/mL of nicotine with 50/50 PG/VG). The cinnamon flavor was purchased from Bio Concept (Niort, France); one drop per mL was added in the e-liquid. The clearomizer was the GS Air 2 Atomizer (Eleaf, Shenzhen, China) with the resistance GS Air Series Atomizer Head 0.75 Ω (Eleaf, Shenzhen, China) and the battery iStick TC40W (Eleaf, Shenzhen, China) always fully charged. We used either a low power (8 W) or a high power (25 W) to generate aerosol condensates. The airflow position was kept open to the maximum for all the experiments. As shown in Figure 1, we defined several experimental conditions by making some parameters vary to assess their influence on the toxicity induced.

The e-liquid from our baseline group consisted of 50/50 PG/VG, i.e., 50% PG and 50% VG, and contained neither nicotine nor flavors. The group exposed to e-liquid + nicotine consisted of the same e-liquid, with 18 mg/mL nicotine added. We chose this nicotine concentration as a recent survey conducted by the French Agency for Food, Environmental and Occupational Health & Safety (ANSES) reported that the average nicotine dose in e-liquid was 6 mg/mL within the French market [24]. However, the proportion of products containing the lowest levels of nicotine (1 to 6 mg/mL) is slightly but constantly decreasing (25–30% to less than 20%) to the benefit of higher nicotine concentration (18 mg/mL) [24]. The aerosol was generated thanks to the Programmable Dual Syringe Pump (PDSP, Burghart Messtechnik GmbH, Wedel, Germany). The aerosol was then collected in a Glass Twin Impinger (GTI, Copley Scientific Limited, Nottingham, UK) filled with 30 mL of PBS in the lower chamber (Erlenmeyer) and corresponds to the aerosol respirable dose. The set up consisted of a GTI connected to a vacuum pump (Low Capacity Pump Model LCP5, flow rate 60 L.min^−1^) manufactured by Copley Scientific. The e-cigarette was held at a 30° angle (to mimic the tilt during a puff), and all the vaping parameters defined by the French Standardization Association (Association Française de normalization, AFNOR standard XP D90-300-3) [25] were respected: 3 sec puff, 55 mL/puff, 30 sec inter-puff, 20 puffs per series, 2 series, 300 sec inter-series. Therefore, a total of 40 puffs were generated to collect the condensates for each condition. The clearomizer was weighed before and after the condensation to evaluate the e-liquid mass in the condensate and thus its concentration. The condensates were then diluted in the culture medium (SmBM, Lonza, Basel, Switzerland) to the desired concentrations. We chose the 2–128 µg/mL concentration range based on the pharmacokinetics of nicotine reported in the literature. Our lowest concentration of nicotine (31.25 ng/mL corresponding to the 2 µg/mL e-liquid concentration) corresponds to a physiological concentration (about the concentration of the peak observed in plasma: 20–40 ng/mL). A large range of concentration was then used (up to 64-fold) to enable us to highlight potential dose-dependent effects (the concentrations in nicotine were 125, 1000 and 2000 ng/mL in the 8, 64 and 128 µg/mL e-liquid concentration, respectively).

### 2.3. Toxicity Assessment

#### 2.3.1. Cytotoxicity

To evaluate the cell membrane damages induced by the exposure of cells to e-liquids or aerosol condensates, the release of lactate dehydrogenase (LDH) in the culture supernatant was assessed after a 24 h incubation, using the CytoTox-96 Assay (Promega, Madison, WI, USA) according to the manufacturer’s instructions. The optical density of the samples was determined using a microplate reader at 490 nm (Multiskan GO, ThermoFisher Scientific, Waltham, MA, USA) and was reported to that of control (unexposed) cells. The positive control consisted of the maximal cellular LDH released (100%) after cell lysis.

#### 2.3.2. Production of Reactive Oxygen Species (ROS)

ROS production was assessed using the OxiSelect ROS Assay Kit (Cell Biolabs, San Diego, CA, USA) following the manufacturer’s instructions. Fluorescence was detected using a fluorometer (Fluoroskan Ascent, ThermoFisher Scientific, Waltham, MA, USA, excitation: 480 nm, emission: 530 nm). The generation of ROS was reported to that of control (unexposed) cells. A positive control consisted of cells exposed to 1 mM hydrogen peroxide.

#### 2.3.3. IL-8 Production

Interleukin-8 (IL-8) production was assessed using the human IL-8 ELISA Kit (ThermoFisher Scientific, Waltham, MA, USA) according to the manufacturer’s instructions. The plate was read with a microplate reader (Multiskan GO, ThermoFisher Scientific, Waltham, MA, USA) set to 450 nm. The production of IL-8 was reported to that of control (unexposed) cells.

### 2.4. Statistical Analyses

The results are expressed as means of 3 independent experiments, each performed in duplicate. Data were treated using Origin and a two-sided Student’s *t*-test was performed to compare experimental groups to the control (unexposed) group. In addition, ANOVA analyses were performed for multiple comparisons (between the different experimental groups). *p* values lower than 0.05 were considered statistically significant.

## 3. Results

### 3.1. Cytotoxicity

**Figure 2:**
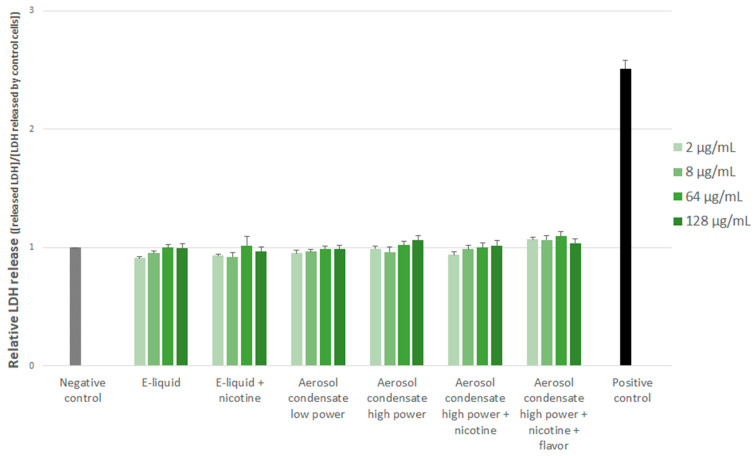
Relative cytotoxicity induced by the different experimental conditions assessed after 24 h of cell exposure by the LDH release. Results are expressed relative to control (unexposed) cells and are means of three independent experiments, each performed in duplicate.

As shown by Figure 2, the LDH release did not vary significantly from that of control cells, whatever the experimental conditions, indicating that neither the e-liquids, nor the aerosol condensates were able to induce cytotoxicity irrespective of their concentrations.

### 3.2. ROS Production

**Figure 3:**
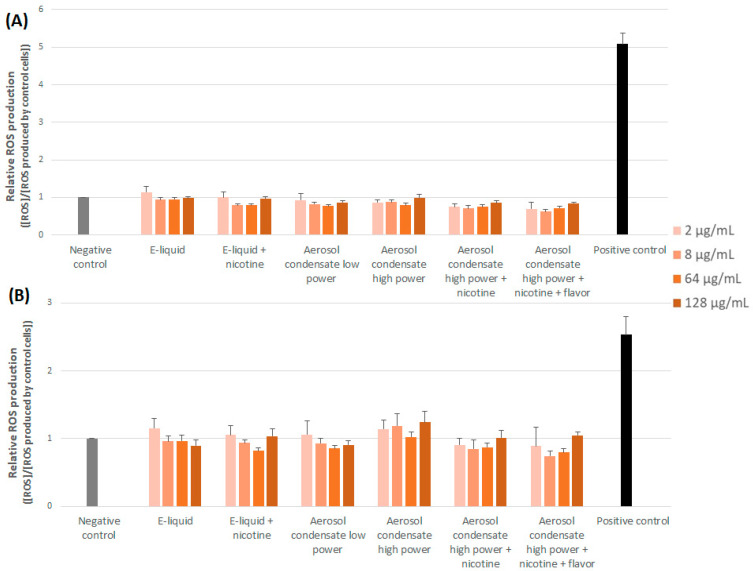
Relative ROS production induced by the different experimental conditions and assessed after 90 min (**A**) or 24 h (**B**) of cell exposure. Results are expressed relative to control (unexposed) cells and are means of three independent experiments, each performed in duplicate.

Figure 3 illustrates the ROS production observed after cell exposure to e-liquids or aerosol condensates. We can clearly observed that it was not significantly enhanced compared to that of control (unexposed) cells, suggesting that no oxidative stress was induced.

### 3.3. IL-8 Production

**Figure 4:**
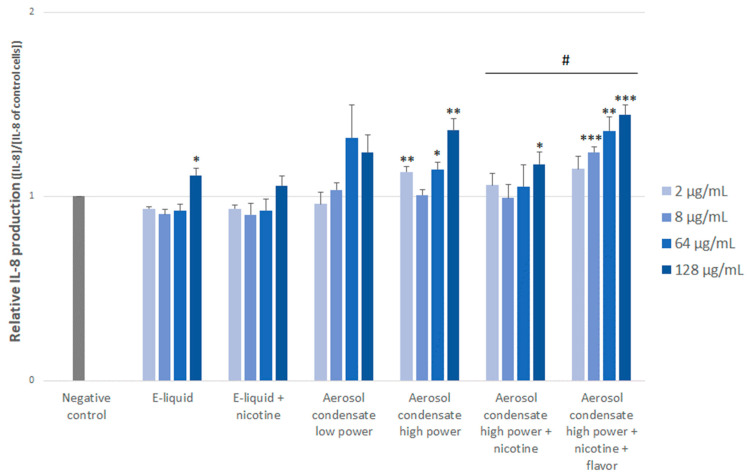
Relative IL-8 production induced by the different experimental conditions and assessed after 24 h of cell exposure. Results are expressed relative to control (unexposed) cells and are means of three independent experiments, each performed in duplicate. Statistical significance is also indicated: * *p* < 0.05, ** *p* < 0.01, *** *p* < 0.001 as determined with a two-sided Student’s *t*-test between control and experimental groups. For inter-group comparisons, an ANOVA analysis was performed: # *p* = 0.024.

Figure 4 depicts the production of the IL-8 pro-inflammatory cytokine. Interestingly, we observed that e-liquid alone induced a slightly significant increase in IL-8 production, only at the highest dose. But we have to keep in mind that this dose is very high and was used to highlight a potential dose-effect. However, no effect of the presence of nicotine could be observed (neither when we compared the e-liquid and e-liquid + nicotine groups (*p* = 0.829) nor when we compared the aerosol condensate high power and aerosol condensate high power + nicotine groups (*p* = 0.309)). Although the difference was not statistically significant (*p* = 0.06), condensates of e-cigarette emission produced with high power induced an enhanced IL-8 production, suggesting that the pro-inflammatory effect of the thermal degradation products (e.g., metals, VOC, aldehydes) was obtained after heating the e-liquid. Finally, the presence of the cinnamon flavor in the aerosol condensate seemed to influence significantly the pro-inflammatory effect of the aerosol condensates, as the aerosol condensate high power + nicotine + flavor condition triggered an IL-8 production higher than its counterpart without flavor (*p* = 0.024).

## 4. Discussion

Our results seem to indicate that a pro-inflammatory response is triggered in aortic smooth muscle cells following exposure to e-cigarette aerosol condensates, when produced at high power and from e-liquid containing cinnamon flavor, suggesting a particular contribution of thermal degradation products and the presence of flavor on e-cigarette toxicity. Conversely, base components of e-liquid only induced a minor IL-8 production, and only at the highest concentration. Similarly, PG/VG alone in e-liquid were shown to affect cell viability in human airway smooth muscle cells at the highest dose [26]. Although PG/VG are “Generally Recognized As Safe” by the FDA, our data show that they could promote a pro-inflammatory response. The impact of PG/VG mixture on IL-8 secretion was further increased in aerosol condensates, suggesting that thermal degradation products could potentiate the inflammatory process.

The presence of nicotine in e-liquids or aerosol condensates did not further enhance the IL-8 production. On the contrary, a slight decrease could be observed compared to nicotine-free samples. Interestingly, human small airway epithelial cells exposed to nicotine-free e-vapor displayed a significantly increased production of IL-6, while nicotine-containing aerosols (with concentrations similar to those used in the present study) tended to decrease IL-6 secretion [27]. This was attributed to the immune suppressive effects of nicotine [28]. Although the experimental conditions were different from ours (epithelial vs. smooth muscle cells and IL-6 vs. IL-8 cytokine), our results suggested in the same way that nicotine was not involved in pro-inflammatory cytokine production in aortic smooth muscle cells. However, the toxicological impact of nicotine remains controversial since another study showed that nicotine enhanced pro-inflammatory effects in airway epithelial cells [29]. Since the role of nicotine is often overlooked against the impact of flavors or devices, further studies are required to clarify the potential harmful effects of nicotine [5].

It has been shown that the main carbonyl compounds found in e-cigarette vapor (formaldehyde, acetaldehyde, and acrolein) result from the thermal decomposition of propylene glycol and glycerin and some flavorings and are known to cause oxidative stress and inflammation [6,30]. Carbonyl compound concentrations are voltage-dependent and increase with high voltage, causing increased thermal degradation products. E-cigarette vapor extracts have been shown to induce a pro-inflammatory response in human neutrophils [18], as well as an increased release of inflammatory mediators from keratinocyte and alveolar epithelial cell lines [31] or alveolar macrophages [32].

On the other hand, flavoring molecules add significant variations to e-cigarette aerosol composition, and, while most are considered as safe when ingested orally, little is known about their systemic effects following inhalation [6]. For example, cinnamaldehyde is considered safe via oral administration; however, inhalation induces dysfunction of pulmonary immune cells through alterations of pro-inflammatory cytokines, a theorized mechanism for adverse cardiac function [33]. Similarly, when exposed to commonly used e-cigarette flavoring chemicals (including cinnamaldehyde), monocytic cells secreted IL-8 chemokine in a dose-dependent manner compared to the unexposed cell groups depicting a biologically significant inflammatory response [34], which is in accordance with our results. In another study, after exposure of human aortic endothelial cells to flavors, expression of the IL-6 pro-inflammatory marker was observed [35]. Endothelial cell dysfunction was also observed when porcine aortic endothelial cells were exposed to cinnamaldehyde [36]. Flavors and especially cinnamaldehyde have also been reported to affect respiratory cells based on oxidative stress and inflammatory processes [37,38,39]. The toxicity induced by cinnamaldehyde could be related to its chemical nature. Indeed, high concentrations of aldehyde flavoring agents in commercially available e-liquids, such as vanillin, ethyl vanillin, benzaldehyde, 4-anisaldehyde (4-methoxybenzaldehyde), cocoa hexenal (5-methyl-2-phenyl-2-hexenal), and cinnamaldehyde, have been reported. Many of them exhibit structural similarity to toxic aldehydes in cigarette smoke [33].

Finally, it is noteworthy, as a reminder, that the findings of this study should be contextualized, and care should be taken to not over-extrapolate conclusions. Indeed, we have chosen to work with human aortic smooth muscle cells because these cells regulate actomyosin involved in contraction and are partly responsible for arterial stiffening, which is an observed clinical effect of e-cigarettes, this is why they can be considered as a relevant model to investigate the cardiovascular toxicity of e-liquids and e-cigarette aerosol condensates. However, they are not directly in contact with blood; we can only hypothesize that some chemicals may diffuse from the blood and reach these cells, albeit concentrations should be low.

To the best of our knowledge, no e-cigarette toxicological studies based on cardiovascular smooth muscle cells have been conducted yet, and only a few studies investigated e-cigarette toxicity on endothelial cells. Interestingly, Anderson et al. reported a limited cytotoxicity of e-cigarette condensate from commercial e-liquids following 24 h incubation with endothelial cells (HUVEC); cytotoxicity significantly increased after 72 h for the highest nicotine concentrations. Similarly, ROS production and DNA damage were increased compared to control following 24 h incubation only at the highest dose [40]. IL-8 production of HUVEC was increased following 24 h incubation with both watermelon and cola flavored e-liquid condensates, while no effects were displayed for other pro-inflammatory cytokines (TNF-α, IL-1β and IL-6) [41]. Finally, a decrease in cell viability and an increased LDH release were observed in HUVEC treated with the highest dose of e-liquid condensates from six commercially available e-liquids following 48 h (cell viability) and 24 h (released LDH) exposure [42]. Although all of these studies strongly suggest that e-liquid condensates could trigger toxicological responses in endothelial cells, the results must be carefully interpreted and compared, since experimental designs were substantially different in terms of puff generation, doses expression, incubation times, and cell models.

## 5. Conclusions

In conclusion, our results suggest that the presence of thermal degradation products (e.g., metals, VOC, aldehydes), as well as the presence of cinnamon flavor, significantly enhanced the pro-inflammatory response of aortic smooth muscle cells when exposed to e-cigarette aerosol condensates, while unheated e-liquid base constituents exerted minor impact. However, these parameters had no influence on cytotoxicity and ROS production. This study deserves further investigation to confirm this finding, especially assessing other cytokines and other flavors. But it paves the way for future research avenues allowing light to be shed on the biological effect of vaping on the cardiovascular system.

## Data Availability

Not applicable.

## References

[1] Hajek P., Phillips-Waller A., Przulj D., Pesola F., Myers Smith K., Bisal N., Li J., Parrott S., Sasieni P., Dawkins L. (2019). A Randomized Trial of E-Cigarettes versus Nicotine-Replacement Therapy. N. Engl. J. Med..

[2] McNeill A., Brose L., Calder R., Simonavicius E., Robson D. (2021). Vaping in England: An Evidence Update Including Vaping for Smoking Cessation, February 2021: A Report Commissioned by Public Health England.

[3] Marques P., Piqueras L., Sanz M.-J. (2021). An Updated Overview of E-Cigarette Impact on Human Health. Respir. Res..

[4] Dinakar C., O’Connor G.T. (2016). The Health Effects of Electronic Cigarettes. N. Engl. J. Med..

[5] Cao Y., Wu D., Ma Y., Ma X., Wang S., Li F., Li M., Zhang T. (2021). Toxicity of Electronic Cigarettes: A General Review of the Origins, Health Hazards, and Toxicity Mechanisms. Sci. Total Environ..

[6] Buchanan N.D., Grimmer J.A., Tanwar V., Schwieterman N., Mohler P.J., Wold L.E. (2020). Cardiovascular Risk of Electronic Cigarettes: A Review of Preclinical and Clinical Studies. Cardiovasc. Res..

[7] Behar R.Z., Wang Y., Talbot P. (2018). Comparing the Cytotoxicity of Electronic Cigarette Fluids, Aerosols and Solvents. Tob. Control.

[8] Wang G., Liu W., Song W. (2019). Toxicity Assessment of Electronic Cigarettes. Inhal. Toxicol..

[9] Vlachopoulos C., Ioakeimidis N., Abdelrasoul M., Terentes-Printzios D., Georgakopoulos C., Pietri P., Stefanadis C., Tousoulis D. (2016). Electronic Cigarette Smoking Increases Aortic Stiffness and Blood Pressure in Young Smokers. J. Am. Coll. Cardiol..

[10] Carnevale R., Sciarretta S., Violi F., Nocella C., Loffredo L., Perri L., Peruzzi M., Marullo A.G.M., De Falco E., Chimenti I. (2016). Acute Impact of Tobacco vs Electronic Cigarette Smoking on Oxidative Stress and Vascular Function. Chest.

[11] Chaumont M., de Becker B., Zaher W., Culié A., Deprez G., Mélot C., Reyé F., Van Antwerpen P., Delporte C., Debbas N. (2018). Differential Effects of E-Cigarette on Microvascular Endothelial Function, Arterial Stiffness and Oxidative Stress: A Randomized Crossover Trial. Sci. Rep..

[12] Franzen K.F., Willig J., Cayo Talavera S., Meusel M., Sayk F., Reppel M., Dalhoff K., Mortensen K., Droemann D. (2018). E-Cigarettes and Cigarettes Worsen Peripheral and Central Hemodynamics as Well as Arterial Stiffness: A Randomized, Double-Blinded Pilot Study. Vasc. Med..

[13] Skotsimara G., Antonopoulos A.S., Oikonomou E., Siasos G., Ioakeimidis N., Tsalamandris S., Charalambous G., Galiatsatos N., Vlachopoulos C., Tousoulis D. (2019). Cardiovascular Effects of Electronic Cigarettes: A Systematic Review and Meta-Analysis. Eur. J. Prev. Cardiol..

[14] Antoniewicz L., Brynedal A., Hedman L., Lundbäck M., Bosson J.A. (2019). Acute Effects of Electronic Cigarette Inhalation on the Vasculature and the Conducting Airways. Cardiovasc. Toxicol..

[15] Caporale A., Langham M.C., Guo W., Johncola A., Chatterjee S., Wehrli F.W. (2019). Acute Effects of Electronic Cigarette Aerosol Inhalation on Vascular Function Detected at Quantitative MRI. Radiology.

[16] Keith R., Bhatnagar A. (2021). Cardiorespiratory and Immunologic Effects of Electronic Cigarettes. Curr. Addict. Rep..

[17] Kuntic M., Oelze M., Steven S., Kröller-Schön S., Stamm P., Kalinovic S., Frenis K., Vujacic-Mirski K., Bayo Jimenez M.T., Kvandova M. (2020). Short-Term e-Cigarette Vapour Exposure Causes Vascular Oxidative Stress and Dysfunction: Evidence for a Close Connection to Brain Damage and a Key Role of the Phagocytic NADPH Oxidase (NOX-2). Eur. Heart J..

[18] Higham A., Rattray N.J.W., Dewhurst J.A., Trivedi D.K., Fowler S.J., Goodacre R., Singh D. (2016). Electronic Cigarette Exposure Triggers Neutrophil Inflammatory Responses. Respir. Res..

[19] Landmesser U., Hornig B., Drexler H. (2004). Endothelial Function: A Critical Determinant in Atherosclerosis?. Circulation.

[20] von Hundelshausen P., Schmitt M.M.N. (2014). Platelets and Their Chemokines in Atherosclerosis-Clinical Applications. Front. Physiol..

[21] Lacolley P., Regnault V., Segers P., Laurent S. (2017). Vascular Smooth Muscle Cells and Arterial Stiffening: Relevance in Development, Aging, and Disease. Physiol. Rev..

[22] Zhuge Y., Zhang J., Qian F., Wen Z., Niu C., Xu K., Ji H., Rong X., Chu M., Jia C. (2020). Role of Smooth Muscle Cells in Cardiovascular Disease. Int. J. Biol. Sci..

[23] Lee W.H., Ong S.-G., Zhou Y., Tian L., Bae H.R., Baker N., Whitlatch A., Mohammadi L., Guo H., Nadeau K.C. (2019). Modeling Cardiovascular Risks of E-Cigarettes With Human-Induced Pluripotent Stem Cell–Derived Endothelial Cells. J. Am. Coll. Cardiol..

[24] ANSES (2020). RAPPORT de l’Anses Relatif à la Déclaration des Produits du Tabac et des Produits Connexes en France—Produits du Vapotage—Bilan 2016–2020.

[25] AFNOR (2021). Norme XP D90-300-3 Cigarettes Electroniques et e-Liquides—Exigences et Méthodes D’essai Relatives aux Emissions (Electronic Cigarettes and e-Liquids—Requirements and Test Methods for Emissions).

[26] Sassano M.F., Davis E.S., Keating J.E., Zorn B.T., Kochar T.K., Wolfgang M.C., Glish G.L., Tarran R. (2018). Evaluation of E-Liquid Toxicity Using an Open-Source High-Throughput Screening Assay. PLoS Biol..

[27] Gellatly S., Pavelka N., Crue T., Schweitzer K.S., Day B.J., Min E., Numata M., Voelker D.R., Scruggs A., Petrache I. (2020). Nicotine-Free e-Cigarette Vapor Exposure Stimulates IL6 and Mucin Production in Human Primary Small Airway Epithelial Cells. JIR.

[28] Kalra R., Singh S.P., Pena-Philippides J.C., Langley R.J., Razani-Boroujerdi S., Sopori M.L. (2004). Immunosuppressive and Anti-Inflammatory Effects of Nicotine Administered by Patch in an Animal Model. Clin. Vaccine Immunol..

[29] Wu Q., Jiang D., Minor M., Chu H.W. (2014). Electronic Cigarette Liquid Increases Inflammation and Virus Infection in Primary Human Airway Epithelial Cells. PLoS ONE.

[30] Merecz-Sadowska A., Sitarek P., Zielinska-Blizniewska H., Malinowska K., Zajdel K., Zakonnik L., Zajdel R. (2020). A Summary of In Vitro and In Vivo Studies Evaluating the Impact of E-Cigarette Exposure on Living Organisms and the Environment. Int. J. Mol. Sci..

[31] Cervellati F., Muresan X.M., Sticozzi C., Gambari R., Montagner G., Forman H.J., Torricelli C., Maioli E., Valacchi G. (2014). Comparative Effects between Electronic and Cigarette Smoke in Human Keratinocytes and Epithelial Lung Cells. Toxicol. In Vitro.

[32] Scott A., Lugg S.T., Aldridge K., Lewis K.E., Bowden A., Mahida R.Y., Grudzinska F.S., Dosanjh D., Parekh D., Foronjy R. (2018). Pro-Inflammatory Effects of e-Cigarette Vapour Condensate on Human Alveolar Macrophages. Thorax.

[33] Clapp P.W., Pawlak E.A., Lackey J.T., Keating J.E., Reeber S.L., Glish G.L., Jaspers I. (2017). Flavored E-Cigarette Liquids and Cinnamaldehyde Impair Respiratory Innate Immune Cell Function. Am. J. Physiol. Lung. Cell. Mol. Physiol..

[34] Muthumalage T., Prinz M., Ansah K.O., Gerloff J., Sundar I.K., Rahman I. (2017). Inflammatory and Oxidative Responses Induced by Exposure to Commonly Used E-Cigarette Flavoring Chemicals and Flavored e-Liquids without Nicotine. Front. Physiol..

[35] Fetterman J.L., Weisbrod R.M., Feng B., Bastin R., Tuttle S.T., Holbrook M., Baker G., Robertson R.M., Conklin D.J., Bhatnagar A. (2018). Flavorings in Tobacco Products Induce Endothelial Cell Dysfunction. Arterioscler. Thromb. Vasc. Biol..

[36] Wölkart G., Kollau A., Stessel H., Russwurm M., Koesling D., Schrammel A., Schmidt K., Mayer B. (2019). Effects of Flavoring Compounds Used in Electronic Cigarette Refill Liquids on Endothelial and Vascular Function. PLoS ONE.

[37] Lerner C.A., Sundar I.K., Yao H., Gerloff J., Ossip D.J., McIntosh S., Robinson R., Rahman I. (2015). Vapors Produced by Electronic Cigarettes and E-Juices with Flavorings Induce Toxicity, Oxidative Stress, and Inflammatory Response in Lung Epithelial Cells and in Mouse Lung. PLoS ONE.

[38] Gerloff J., Sundar I.K., Freter R., Sekera E.R., Friedman A.E., Robinson R., Pagano T., Rahman I. (2017). Inflammatory Response and Barrier Dysfunction by Different E-Cigarette Flavoring Chemicals Identified by Gas Chromatography-Mass Spectrometry in e-Liquids and e-Vapors on Human Lung Epithelial Cells and Fibroblasts. Appl. In Vitro Toxicol..

[39] Kaur G., Muthumalage T., Rahman I. (2018). Mechanisms of Toxicity and Biomarkers of Flavoring and Flavor Enhancing Chemicals in Emerging Tobacco and Non-Tobacco Products. Toxicol. Lett..

[40] Anderson C., Majeste A., Hanus J., Wang S. (2016). E-Cigarette Aerosol Exposure Induces Reactive Oxygen Species, DNA Damage, and Cell Death in Vascular Endothelial Cells. Toxicol. Sci..

[41] Su L., Zhao M., Ma F., An Z., Yue Q., Zhao C., Sun X., Zhang S., Xu J., Jiang X. (2022). A Comparative Assessment of E-Cigarette Aerosol Extracts and Tobacco Cigarette Smoke Extracts on in Vitro Endothelial Cell Inflammation Response. Hum. Exp. Toxicol..

[42] De Martin S., Gabbia D., Bogialli S., Biasioli F., Boschetti A., Gstir R., Rainer D., Cappellin L. (2021). Refill Liquids for Electronic Cigarettes Display Peculiar Toxicity on Human Endothelial Cells. Toxicol. Rep..

